# Supplementary material for: Design and validation of a GMP stem cell manufacturing protocol for MPSII hematopoietic stem cell gene therapy
Source: Mol Ther Methods Clin Dev. 2024 May 21;32(2):101271. doi: 10.1016/j.omtm.2024.101271 (PMC11214401; doi:10.1016/j.omtm.2024.101271)
Supplement: Document S1. Tables S1‒S6 [file mmc1.pdf]

OMTM, Volume *32*

Supplemental information

Design and validation of a GMP stem cell manufacturing protocol for MPSII hematopoietic stem cell gene therapy

Stuart Ellison, Karen Buckland, Yuko Learmonth, Victoria Day, Spandan Kalra, Lauren Howe, Francisco José Roman-Rodriguez, Jose Bonafont, Laura Booth, Rebecca Holley, Jon Smythe, Simon Jones, Adrian Thrasher, Claire Booth, and Brian W. Bigger

Table S1. Current specification for batch release testing of a cryopreserved medicinal product for proposed clinical trial

Test	Method	Acceptance Criteria
Mycoplasma	PCR	Not Detected
Total Viable Cell Count/ml	Trypan Blue	0.5 to 17.5 x 10^6 TNC/ml
Total Viable Cell Count	Trypan Blue	Record
CD34+ Cell Purity and Identity	FACS	≥ 35% CD34+ cells
CD34+ Cell Dose	Calculated	Record
Cell Viability	Trypan Blue	≥ 70% viable cells
Sterility	BacTec	No Growth
Bacterial Endotoxins	Kinetic Turbidimetric	≤ 5 EU/kg/hr
Vector Copy Number	ddPCR	≥ 0.5 copy/cell

Table S2: Small scale hCD34+ stem cell transduction optimisation performed at GOSH. Low vector dose range.

Methodology	Batch	MOI	Vector Conc. (x 10^8 IG/ml)	Healthy Donor Cells	Batch Testing					
					**Test Attribute**	**Viability**	**CD34%**	**Clono-genicity**	**VCN (liquid culture)**	**Pass/ Fail**
					Method	Trypan Blue	Flow Cytometry	CFU assay	ddPCR	
					Acceptance Criteria	≥ 70%	For information only	≥ 4 CFUs/ 1000 cells plated	≥ 0.5	
GMP Experiment	21-SU-MPSII-01a	25	0.5	A		94.01	99.2	366	0.93	Pass
		12.5	0.25	A		92.87	99.0	346	0.85	Pass
		25	0.5	B		93.01	99.1	406	0.839	Pass
		12.5	0.25	B		92.94	99.2	374	0.827	Pass
		N/A	N/A	A		92.87	99.0	336	0.01	N/A
		N/A	N/A	B		99.6	99.6	398	0	N/A

Table S3: Small scale hCD34+ stem cell transduction optimisation performed at GOSH. High vector dose range.

*A minimum of 3.0 x 10^6 CD34+ cells/kg after transduction is required for infusion into the patient

Methodology	Batch	MOI**	Vector Conc. (x 10^8 IG/ml)*	Healthy Donor Cells	Batch Testing					
					**Test Attribute**	**Viability**	**CD34%**	**VCN (liquid culture)**	**VCN (pooled CFUs)**	**Pass/ Fail**
					Method	Trypan Blue	Flow Cytometry	ddPCR	ddPCR	
					Acceptance Criteria	≥ 70%	For information only	≥ 0.5	≥ 0.5	
GMP Experiment	21-SU-MPSII-01b	25	0.5	A		91.6	99.3	0.92	1.1	Pass
		50	1.0	A		89.13	99.5	1.19	1.08	Pass
		100	2.0	A		92.46	99.0	1.63	1.56	Pass
		25	0.5	C		91.07	99.2	0.99	1.5	Pass
		50	1.0	C		95.03	99.5	1.08	1.27	Pass
		100	2.0	C		92.53	99.5	1.92	2.09	Pass

*Cells from the same health donor A were used in both assays (table S2 and S3).**MOI = Multiplicity of infection

Table S4: UoM/Barnsley vs GOSH equivalent vector concentration comparison.

UoM/Barnsley MOI	GOSH MOI
25	18
50	35
100	71

Table S5. Individual VCN counts in single picked colonies from CFU assay, determined by QPCR. Left panel) Large scale transduction performed at Manchester research lab with LV IDS.ApoEII at MOI25 + TE with IL3 in the media, cultured in rectronection-coatedT175 flask. Right panel) 'At scale' transduction performed at NHSBT Barnsley research and development lab with LV IDS.ApoEII at MOI25 + TE with IL3 in the media, cultured in rectronection-coated T175 flask.

BFU-E	CFU-GM		BFU-E	CFU-GM
1.52	1.51		1.41	28.00**
5.96	6.42		1.89	1.10
1.90	1.88		1.34	6.28
4.92	4.49		2.26	10.24
3.86	1.35		1.25	8.43
3.56	5.73		2.69	3.51
1.51	4.55		4.27	2.41
6.42	2.76		10.20	8.07
1.88	1.31		0.15*	5.53
4.49	1.29		7.26	4.62
1.35	7.88		5.63	2.80
5.73	1.62		2.21	5.26
			3.58	4.07
			2.49	3.32
			4.06	0.82
			3.21	3.30
			1.28	-

* VCN below 0.3 classed as 0 (colony with no copies).
**VCN assay is accurate up to approximately 12 vector copies. VCNs above this cannot be accurately determined with this method.

Table S6. Overview of sampling timepoints and volumes taken for sterility testing performed for the NHSBT GMP validation runs.

Reagent / Sample	No. of Replicates/micro-organism	Inoculum volume per broth	Comments
Final product – CD11b-IDS.ApoEII LV Gene Modified Autologous CD34+ Cells in final container and condition as presented to patient	3	600µl	Cryopreserved in pilot vial 1% of final product volume
Fresh HPC Apheresis as collected from donor	1	2-4ml	1% of starting material volume depending on received volume
CD34- cell fraction post selection	1	5ml	1% of total selected cell product volumes (+ and – fractions)
Day 3 transduction media supernatent	1	5ml	>1% of total supernatant volume
Day 4 transduction media supernatent	1	5ml	>1% of total supernatant volume
Day 4 wash buffer supernatent	1	5ml	>1% of total supernatant volume
