# Supplementary material for: Design and validation of a GMP stem cell manufacturing protocol for MPSII hematopoietic stem cell gene therapy
Source: Mol Ther Methods Clin Dev. 2024 May 21;32(2):101271. doi: 10.1016/j.omtm.2024.101271 (PMC11214401; doi:10.1016/j.omtm.2024.101271)
Supplement: Document S2. Article plus supplemental information [file mmc2.pdf]

Molecular Therapy
Methods & Clinical Development
Original Article

Design and validation of a GMP stem cell manufacturing protocol for MPSII hematopoietic stem cell gene therapy

Stuart Ellison,[1] Karen Buckland,[3,5] Yuko Learmonth,[1] Victoria Day,[2] Spandan Kalra,[2] Lauren Howe,[2] Francisco José Roman-Rodriguez,[3,5] Jose Bonafont,[3,5] Laura Booth,[1] Rebecca Holley,[1] Jon Smythe,[2] Simon Jones,[4] Adrian Thrasher,[3] Claire Booth,[3,5] and Brian W. Bigger[1,6]

[1]Stem Cell & Neurotherapies Group, University of Manchester, Manchester, UK; [2]Cellular and Molecular Therapies, NHSBT Barnsley, Barnsley, UK; [3]UCL Great Ormond Street Institute of Child Health, London, UK; [4]Manchester University NHS Foundation Trust, Manchester, UK; [5]Great Ormond Street Hospital Biomedical Research Centre, London, UK; [6]Institute for Regeneration and Repair, University of Edinburgh, Edinburgh, UK

Hematopoietic stem cell gene therapy (HSCGT) is a promising therapeutic strategy for the treatment of neurodegenerative, metabolic disorders. The approach involves the *ex vivo* introduction of a missing gene into patients' own stem cells via lentiviral-mediated transduction (TD). Once transplanted back into a fully conditioned patient, these genetically modified HSCs can repopulate the blood system and produce the functional protein, previously absent or non-functional in the patient, which can then cross-correct other affected cells in somatic organs and the central nervous system. We previously developed an HSCGT approach for the treatment of Mucopolysaccharidosis type II (MPSII) (Hunter syndrome), a debilitating pediatric lysosomal disorder caused by mutations in the iduronate-2-sulphatase (IDS) gene, leading to the accumulation of heparan and dermatan sulfate, which causes severe neurodegeneration, skeletal abnormalities, and cardiorespiratory disease. In HSCGT proof-of-concept studies using lentiviral IDS fused to a brain-targeting peptide ApoEII (IDS.ApoEII), we were able to normalize brain pathology and behavior of MPSII mice. Here we present an optimized and validated good manufacturing practice hematopoietic stem cell TD protocol for MPSII in preparation for first-in-man studies. Inclusion of TEs LentiBOOST and protamine sulfate significantly improved TD efficiency by at least 3-fold without causing adverse toxicity, thereby reducing vector quantity required.

INTRODUCTION

Over the past decade, the hematopoietic stem cell gene therapy (HSCGT) approach has proved to be an effective, well-tolerated, and safe treatment strategy for several congenital immunodeficiencies, hematological disorders, and metabolic diseases.[1–3] Hundreds of patients have received HSCGT treatment in clinical trials for conditions such as adenosine deaminase severe combined immunodeficiency (ADA-SCID), SCID-X1, β-thalassemia, chronic granulomatous disease (CGD), X-linked adrenoleukodystrophy, metachromatic leukodystrophy (MLD), and mucopolysaccharidosis type IIIA (MPSIIIA), with the vast majority demonstrating positive clinical benefit.[4–10] As a result, substantial commercial interest has paved the way for advanced therapeutic medicinal products (ATMPs) to be developed for several of these conditions, gaining regulatory approval by the European Medicines Agency in the form of Strimvelis for ADA-SCID, Zynteglo for β-thalassemia, and Libmeldy for MLD,[11–13] with more approved treatments expected to follow in the coming years.

The HSCGT approach involves harvesting hematopoietic stem cells (HSCs), that express the cell surface marker CD34, from apheresis of mobilized stem cells from peripheral blood.[14] To mobilize stem cells into the peripheral blood, patients are typically given a combination of granulocyte-colony stimulating factor (G-CSF) and plerixafor, a small molecule bicyclam CXCR4 antagonist (although the drug combination can vary depending on disease-specific factors), and a leukapheresis performed to harvest the cells.[15] Under aseptic conditions, $CD34^+$ cells are then isolated and undergo a pre-stimulation step in preparation for *ex vivo* transduction (TD), typically two TDs with a lentiviral vector (LV) at a high multiplicity of infection (MOI), to introduce correct copies of the defective gene responsible for the condition.[16] The harvested gene-modified cells, the investigational medicinal product (IMP), then undergoes various quality control (QC) checks before it can be administered back to the patient. The product can be given fresh; however, it is more commonly cryopreserved until needed, while the patient undergoes a conditioning regime, typical for many of the conditions being treated using this approach.[17] Once genetically modified HSCs are infused into the patient, they home to the bone marrow where they engraft in the niche afforded by conditioning, maintaining their self-renewing capacity.

Received 5 February 2024; accepted 17 May 2024;
https://doi.org/10.1016/j.omtm.2024.101271.

Correspondence: Brian W. Bigger, Institute for Regeneration and Repair, 5 Little France Drive, Edinburgh Bioquarter, University of Edinburgh, Edinburgh, UK.
E-mail: brian.bigger@ed.ac.uk

This is an open access article under the CC BY license (http://creativecommons.org/licenses/by/4.0/).

From here, HSC progeny can differentiate into all immune-hematopoietic lineages, passing on the genetic correction as cells divide.[18] Crucially for the treatment of neurological disorders such as MLD and MPSIIIA, HSC progeny can differentiate into monocytes, traffic to the brain, further differentiate into microglial-like cells, and secrete functional enzymes for uptake by affected brain cells in a process called cross-correction.[19]

We have developed an HSCGT treatment for MPSII, a rare, X-linked lysosomal storage disease caused by a defective iduronate-2-sulfatase (IDS) gene (GenBank: NM_000202). MPSII typically affects males, with an incidence of approximately 1.3 per 100,000 live births and disease onset between 2 and 4 years of age.[20,21] Absence of functional IDS enzyme results in the accumulation of undegraded heparan sulfate and dermatan sulfate in the body, causing a range of symptoms including skeletal abnormalities, joint pain, short stature, cardiorespiratory disease, hepatosplenomegaly, and, in severe forms of MPSII, neurodegeneration.[22] We have completed proof-of-concept studies in the MPSII murine model demonstrating efficacy and safety of a brain-targeted HSGCT approach using lentiviral IDS fused to the blood-brain barrier-crossing peptide ApoEII (IDS.ApoEII).[23,24] Sustained IDS enzyme activity was observed in the organs of IDS.ApoEII-treated MPSII mice, with continued clearance of storage material in the brain and peripheral organs, correction of astrogliosis and microgliosis, and correction of altered cytokines and chemokines, with no observed toxicity after treatment, making this therapy an excellent candidate for translation to the clinic.[23–25]

In preparation for a phase 1/2 HSCGT clinical trial in MPSII patients, we have developed, optimized, and validated a clinical stem cell manufacturing process that we report here. We have optimized the LV TD process with the inclusion of TD enhancers (TEs), LentiBOOST and protamine sulfate, in addition to testing different culture vessels and media constituents during scale up validations. Furthermore, we have validated the manufacturing process in a new cleanroom facility in the UK, thereby expanding ATMP manufacturing capacity in the UK in preparation for future HSCGT trials.

RESULTS

TEs significantly improve TD efficiency and efficacy at low vector concentrations

An overview of the HSCGT treatment strategy for MPSII is detailed in Figure 1, with the focus of this work, the manufacture of genetically modified patient cells, highlighted in blue. LV made to GMP standard is required to develop, optimize, and validate a clinical stem cell TD protocol. For this study, GMP-grade IDS.ApoEII LV was manufactured by Indiana University Vector Production Facility.[26] We performed TD optimization studies using cryopreserved human $CD34^+$ cells (hCD34), previously isolated from a healthy donor leukapheresis unit by magnetic bead separation using a CliniMACSplus instrument.[27] We compared a range of IDS.ApoEII LV concentrations (12.5, 25, 50, and 100 MOI) with and without the TEs LentiBOOST and protamine sulfate, as tested in similar TD optimization studies, albeit with different LVs.[28,29] The growth media chosen for these studies was serum-free X-VIVO-15 with the inclusion of 1% human albumin serum (HAS) and fms-related tyrosine kinase 3 ligand (Flt3-L), stem cell factor (SCF), thrombopoietin (TPO) and interleukin-3 (IL-3), as used in a number of GMP cell manufacturing protocols for ADA and CGD.[5,10,30,31] Transduced or mock-transduced cells were seeded in the colony-forming unit (CFU) assay and a number of parameters evaluated after 14 days in culture. The number of burst-forming unit-erythroid (BFU-E), CFU granulocyte, macrophage (GM), and CFU granulocyte, erythrocyte, monocyte, megakaryocyte (GEMM) colonies were counted for each condition to assess lineage development (Figure 2A). In the absence of any LV, there seemed to be a degree of toxicity from TEs alone, with a 40%–50% decrease in colony numbers across the three colony types (140 vs. 73 for BFU-E, 127 vs. 73 for CFU-GM, and 21 vs. 12 for CFU-GEMM). At MOI of 12.5 and 50 + TEs, there were no significant differences in colony numbers compared with the same MOIs without TEs (95 vs. 99, 124 vs. 134 BFU-Es at MOI 12.5, and 50 respectively; 98 vs. 105, 118 vs. 105 CFU-GMs at MOI 12.5 and 50, respectively) and these numbers were comparable with non-transduced cells without TEs. At MOI 25 we observed a reduction in colonies in the presence of TEs, but we suspect this may be down to variability in the assay rather than toxicity, as reduced numbers were not observed at the higher MOI of 50 + TEs (Figure 2A). At the highest LV concentration, MOI of 100 + TEs, it was evident there was toxicity as we observed less than 10 combined BFU-E and CFU-GM colonies and no identifiable CFU-GEMMs across three methylcellulose dishes.

We observed that TEs were particularly effective at enhancing TD efficiency at low LV concentrations meaning a greater proportion of cells receive the insert (Figure 2B). At MOI 12.5, we observed an increase from 33.3% to 94.1% TD of BFU-Es and from 55.6% to 94.1% in CFU-GM colonies, and at MOI 25 an increase from 72.2% to 82.4% TD of BFU-Es and from 61.1% to 94.1% in CFU-GM colonies (Figure 2B). This was consistent with an overall increase in vector copy number (VCN) across all vector concentrations of between 2.5- and 2.9-fold for pooled CFU colonies and cells grown in 14-day liquid culture (LC) (Figure 2C). Similarly, intracellular IDS activity increased on average by 4.8-fold in pooled CFUs and 2.1-fold in LCs seeded from cells transduced with TEs (Figure 2D). Comparing VCN vs. IDS activity in pooled CFUs there was an exponential correlation, compared with a positive linear correlation in LCs (Figures 2E and 2F). These small-scale TD studies confirmed that inclusion of TEs in the TD media can significantly improve TD of $hCD34^+$ cells with IDS.ApoEII LV at low vector concentrations.

Optimized TD conditions can be effectively scaled for transducing larger $hCD34^+$ cell numbers required for clinical application

Following TD optimization studies performed at small scale, we took forward vector concentrations of MOI 12.5 and MOI 25 with the inclusion of TEs and evaluated them in at-scale TDs using larger $CD34^+$ stem cell quantities more relevant to clinical application. First, we

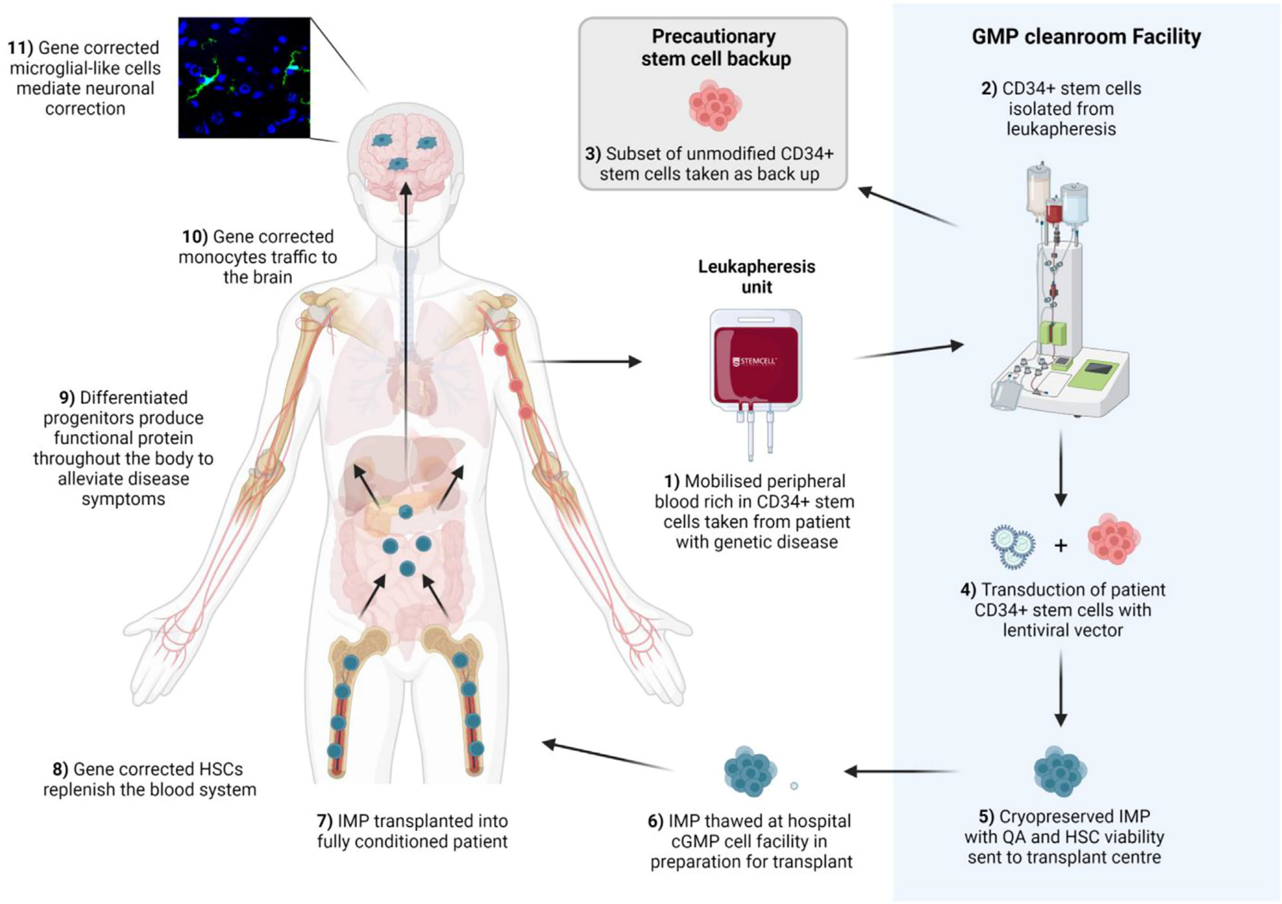

Figure 1. HSCGT overview

The patient is given recombinant human granulocyte colony stimulating factor by subcutaneous injection (G-CSF; 5–16 μg/kg per day for 5–6 days) to mobilize HSPC from the bone marrow (BM) into the peripheral circulation. On the last day, plerixafor maybe given to maximize mobilization. $CD34^+$ cell counts are monitored from day 3 and leukapheresis performed on days 5 and 6 provided cell count is greater than 1×10^4 $CD34^+$ cells/mL. Following leukapheresis, $CD34^+$ cells are isolated by magnetic bead separation and a back-up of a minimum of 3×10^6 $CD34^+$ cells/kg cryopreserved for potential stem cell transplant rescue in the event that engraftment of the IMP fails. The main bulk of selected $CD34^+$ stem cells are pre-stimulated overnight in growth media plus cytokines prior to TD with GMP LV. The following day, the transduced cells are harvested and the majority cryopreserved to make the IMP, with a subset sent for quality assurance (QA) analysis to confirm sterility, absence of mycoplasma and endotoxins, $CD34^+$ cell purity and viability, normal progenitor development in the CFU assay, number of integrated VCNs, and, depending on the specific product, protein activity. The patient undergoes myeloablative conditioning before the IMP can be administered. Following transplant, the gene-corrected HSCs replenish the blood system. Differentiated progenitors can distribute throughout the body, including the brain, and produce functional protein to alleviate the disease phenotype.

performed two test runs of the $CD34^+$ isolation procedure, using a CliniMACS plus instrument,[32,33] on healthy donor-derived leukapheresis units supplied by BIOIVT (USA-based supplier) and the UK Anthony Nolan trust (AN), to establish the typical number and viability of $CD34^+$ cells that could be isolated from a standard unit. We isolated 1.23×10^8 and 1.17×10^8 $CD34^+$ cells from the BIOIVT and AN leukapheresis units respectively, with post-selection $CD34^+$ cell viabilities of greater than 97% as determined by fluorescence-activated cell sorting (FACS) and hemacytometer counts. We opted to source leukapheresis units from AN for all subsequent validation work, as we could receive the product on day of donor collection (compared with 48-h shipment from the United States for the BIOIVT product), ensuring a fresher product with greater longevity, thereby allowing some flexibility with timings for initiation of $CD34^+$ stem cell selection.

To refine the manufacturing process and draft an initial batch manufacturing record (BMR) in preparation for translation to GMP, we performed a pilot TD run in the Manchester University research laboratory. We transduced 8.9×10^6 $CD34^+$ cells (thawed cryopreserved $CD34^+$ product [BIOIVT]) with research grade IDS.ApoEII LV at an MOI of 60 without TEs (an LV concentration used in our previous proof-of-concept murine studies[24,34]), including a mock transduced group as a control. We achieved a VCN of 1.83 in pooled CFUs seeded from the transduced product with an IDS activity of 780 μM 4-MU/4 h/mg protein (Figures 3D–3F). We next

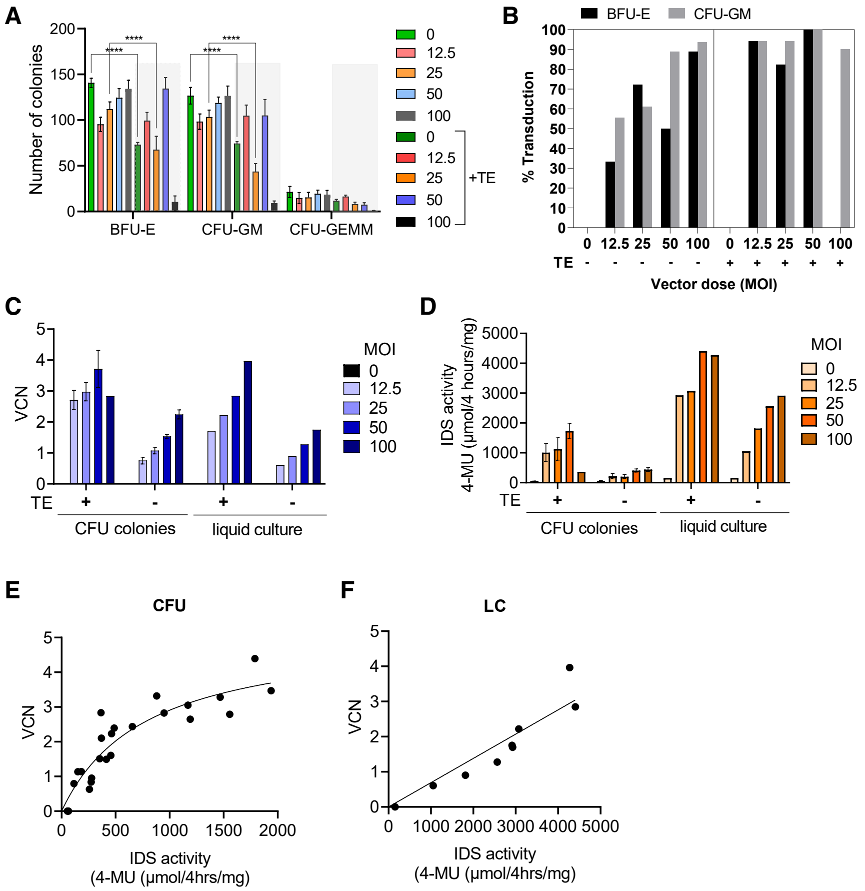

Figure 2. Small scale h CD34+ stem cell TD optimization

(A) CD34+ stem cells were transduced with and without TEs (±TE) at a range of vector concentrations (MOI) and then seeded in 14-day CFU assays and the number of BFU-E, CFU-GM and CFU-GEMM colonies assessed. Data are mean ± SEM. One-way ANOVA. ****$p < 0.0001$. (B) TD efficiency was determined by picking 18 individual BFU-E and CFU-GM colonies for each condition, extracting gDNA and determining if the colonies are positive for the integrated genome by qPCR. (C) The VCN was determined in 14 day pooled CFU or LCs by qPCR. (D) IDS activity in 14-day pooled CFU or LCs. VCN vs. IDS activity in pooled CFU (E) and LCs (F).

performed a complete manufacturing validation run, including a CD34+ cell selection and TD with our optimized concentrations of MOI 12.5 and 25 + TEs with GMP IDS.ApoEII LV (7.5×10^6 CD34+ cells per group). CD34+ cell purity and viability was assessed post-selection, after pre-stimulation and after TD by FACS and all were above 98% (Figure 3A). Overall TD efficiency was 100% for both concentrations (Figure 3B) and CFU colonies followed a normal lineage development pattern (Figure 3C). VCN at the lower concentration of MOI 12.5 + TE in pooled CFUs was 1.9, equivalent to the previous, approximately 5-fold, higher LV concentration of MOI 60 tested without TEs (Figure 3D). An MOI of 25 increased the VCN to 2.7 (Figure 3D). LC VCNs were 3.84 and 4.66 for MOI 12.5 and 25, respectively. Intracellular IDS activity increased following an increase in vector concentration as expected (Figures 3E and 3F). This at-scale study demonstrated effective scale up of the manufacturing process and allowed for development of the BMR required for GMP working.

Process development in preparation for GMP validations

We continued process development in a new, recently opened clean room facility at NHSBT Barnsley UK and performed a pilot manufacturing run to further optimize the manufacturing and cryopreservation process ahead of full-scale GMP validations. This run was used to define QC sampling timepoints and to confirm accurate quantities of reagents and consumables to define accurate picking lists for the BMR. In addition, the product produced during the development run was used to validate various QC tests, including sterility, endotoxin, and mycoplasma assays performed at internal and external laboratories and developed specifically for this manufacturing process to European Pharmacopeia standards. For the pilot run, we also evaluated whether the exclusion of IL-3 from the TD media had any noticeable effect on product viability and efficacy. Many clinical TD protocols include IL-3 in the TD media[30,31]; however, some recent trials have moved away from using IL-3 in the TD media, as it has been suggested it could have a negative impact on engraftment.[28,35,36] Following magnetic bead separation, 8×10^6 CD34+ cells were transduced with GMP IDS.ApoEII LV for each condition in either a retronectin-coated T75 flask with IL-3 or without IL-3. A subset of each product from the last wash step and from the infusion buffer were seeded in 14-day CFU assay and the remainder cryopreserved and assigned for QC testing. Exclusion of IL-3 from the media resulted in a small reduction in colony numbers and TD efficiency (Figures 4A–4C). In the flask with IL-3, we observed an overall TD efficiency of 97.2% compared with 86.1% in the flask without IL-3 (Figure 4C). Exclusion of IL-3 from the TD media also had a significant impact on overall VCN, with a reduction of 2.4-fold observed in the 14-day LC (3.4 vs. 1.4) (Figure 4D), with a similar pattern observed in the pooled CFU assay colonies (3.5 vs. 0.96 and 2.63 vs. 1.43) (Figures 4E and 4F).

GMP validation runs

We next performed two full-scale GMP validations runs in the NHSBT cleanroom. From the pilot study data, we chose to move forward with a vector concentration of MOI 25 + TE with IL-3 and using rectronectin-coated T175 culture flasks. Figure 5 shows a schematic of the finalized manufacturing process validated at NHSBT Barnsley. CFU assays were performed on the product at various stages including

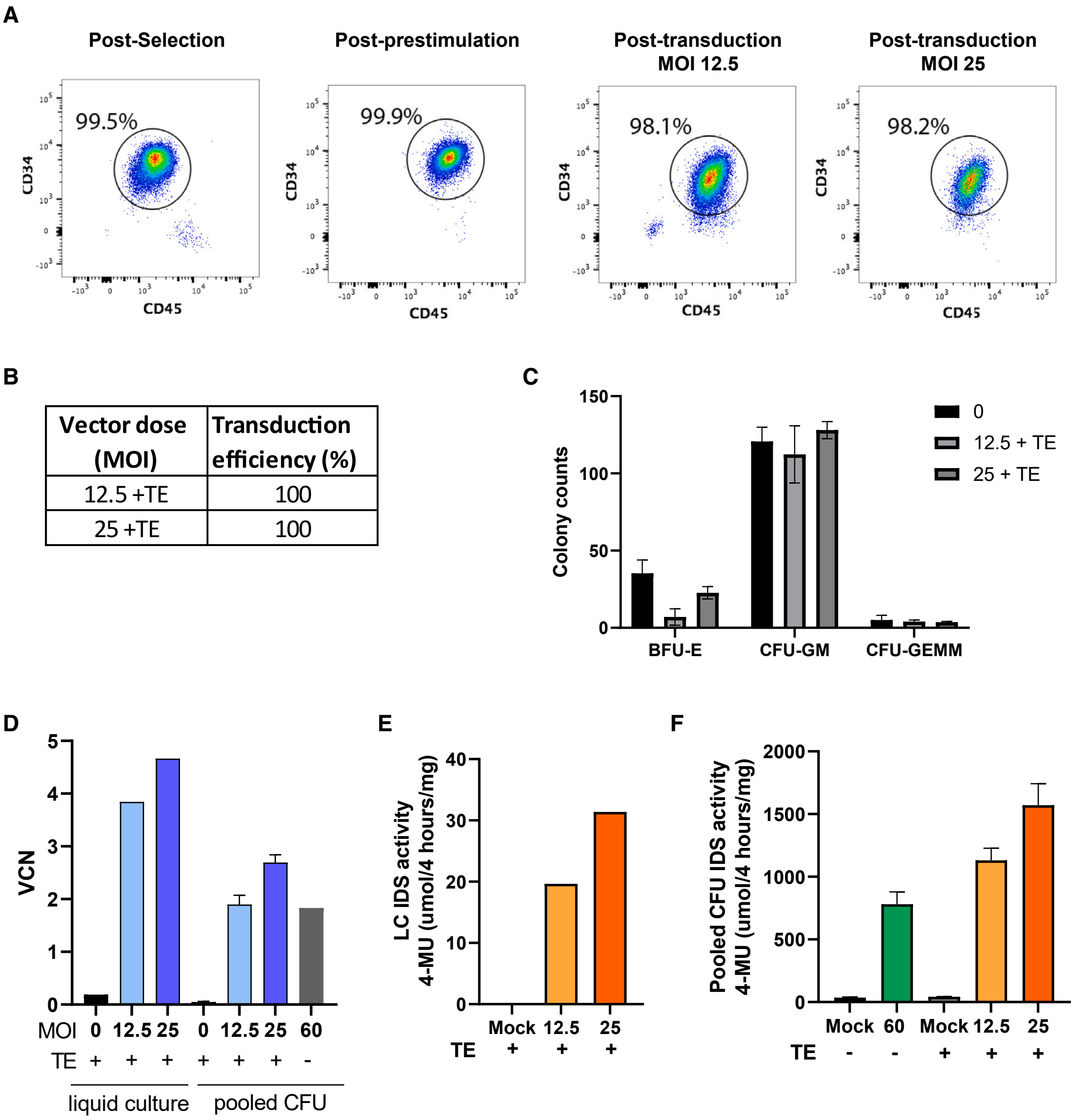

Figure 3. Scale up of hCD34$^+$ stem cell TD

(A) CD34$^+$ stem cell viability was evaluated by FACS post-selection, post-prestimulation and post-TD at MOI 12.5 and 25. Following 14-day CFU assay TD efficiency (B) and number and type of CFU colonies assessed (C). VCN (D) and IDS activity was assessed in liquid (E) and pooled CFU cultures (F). Data are mean ± SEM.

pre-cryopreservation and at 24 h, 6 weeks, and 12 weeks post-thaw time points to provide some stability data. LC assays were performed on the post-thaw 12-week product, following optimization of the protocol at earlier time points. For both runs, we achieved high CD34$^+$ cell viability of 94.8% or above. For run 1, we recovered 127 $\times$ 10^6/kg transduced CD34$^+$ cells with 88% overall recovery from cells seeded and for run 2, 104 $\times$ 10^6/kg transduced CD34$^+$ cells and 88.2% recovery (Table 1). Twenty-four milliliters of cryopreserved product was sent to the Scottish National Blood Transfusion Service for validation of the QA approved sterility assay from run 1, accounting for the lower percentage overall recovery in the post-thaw samples (Table 1). VCN in pooled CFUs ranged between 6.27 and 7.55 for run 1 and between 5.65 and 6.62 for run 2 (Table 1). VCN of the LC was 5.09 and 6.34 for runs 1 and 2, respectively. The final products were absent of mycoplasma, sterile and endotoxin levels were below 0.1 EU/mL meeting release criteria specification (Table S1). CFU counts from the pre-cryopreserved cells indicated slightly reduced colonies numbers post-TD compared with pre-TD cells (203 vs. 274) with a normal pattern of lineage development (Figures 6A and 6E). CFU assays performed at 6 and 12 weeks show BFU-E, CFU-GM, and CFU-GEMM colony numbers comparable

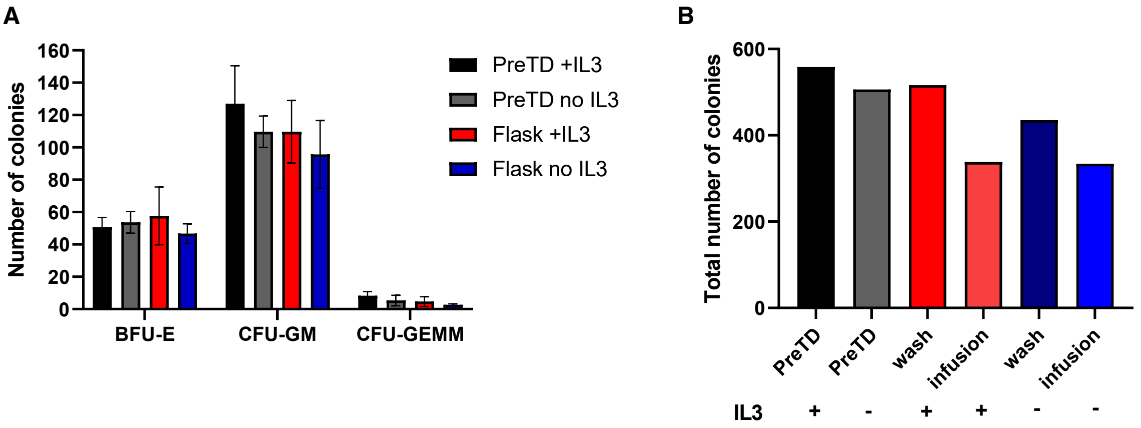

C

Vessel	IL-3	BFU-E	CFU-GM	combined colonies
Flask	+	94.4	100	97.2
Flask	-	72.2	100	86.1

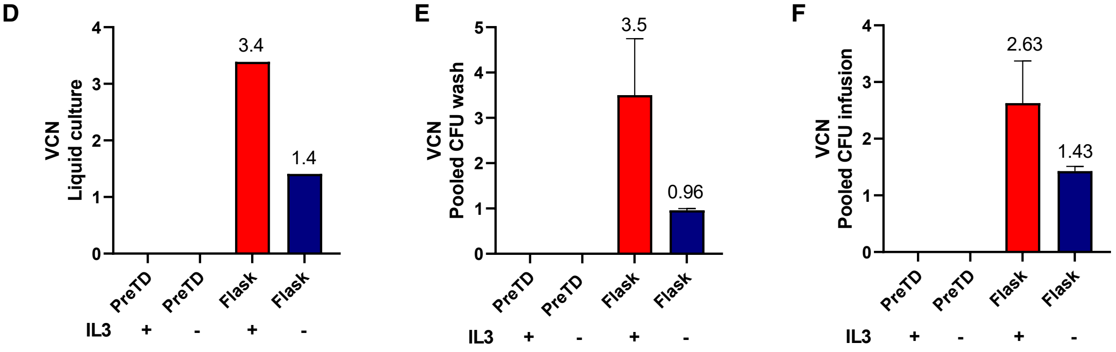

Figure 4. R&D pilot manufacturing run comparing culture vessel and evaluating absence of IL3 in the TD media

$CD34^+$ stem cells were seeded in a T75 flask containing culture media with or without IL3. Cells were transduced for 24 h before being harvested.
(A) Cells taken after the final wash and from the infusion buffer were seeded in the CFU assay and BFU-E, CFU-GM and CFU-GEMM colonies counted 14 days later. (B) Total combined colony counts for each condition. (C) TD efficiency evaluated in BFU-E, CFU-GM, and combined colonies. VCN in LCs (D), in pooled CFUs from final wash (E) and infusion media (F). Data are mean ± SEM.

with pre-cryopreserved cells (Figures 6B, 6C, 6F, and 6G). For LC assays performed at 24 h and 6 weeks after cryopreservation, we observed high cell death (>50%, data not shown), prompting a change in protocol to include twice weekly media changes. We used this refined protocol to perform the LC assay on the 12-week cryopreserved products for runs 1 and 2. Cell viability was much improved over the 2 weeks, leveling out to 69% and 66%, respectively, for runs 1 and 2 by days 12–14 (Figures 6D and 6H).

In parallel to GMP validations being performed at NHSBT Barnsley, GMP validations runs were also being completed at the Great Ormond Street Hospital (GOSH) cleanroom using a similar manufacturing process, and the findings are presented in Table 3. The most notable differences to highlight in the manufacturing process are the exclusion of IL-3 from the media and the use of SCGM (Cellgenix) culture media instead of X-VIVO 15 (Lonza) (Table 2). Lower VCNs were observed in pilot studies with these culture conditions; therefore, a higher vector concentration of MOI100 + TEs was implemented (Tables S2 and S3). Important to note here is that the LV concentrations were calculated based on an LV titer evaluated independently at the GOSH site using a slightly different titration method and thus determined to be 1.4×10^9 TU/mL (compared with 1×10^9 TU/mL determined at the University of Manchester). Therefore, at equivalent MOIs, the vector concentration is approximately two-thirds lower for TDs performed at the GOSH site (Table S4). Cell viability for the transduced product (DP) on day 3 was 91.79 and 91.63% for run 1 and run 2, respectively (Table 3). In the 14-day LC, VCNs were 3.3 and 2.25 for runs

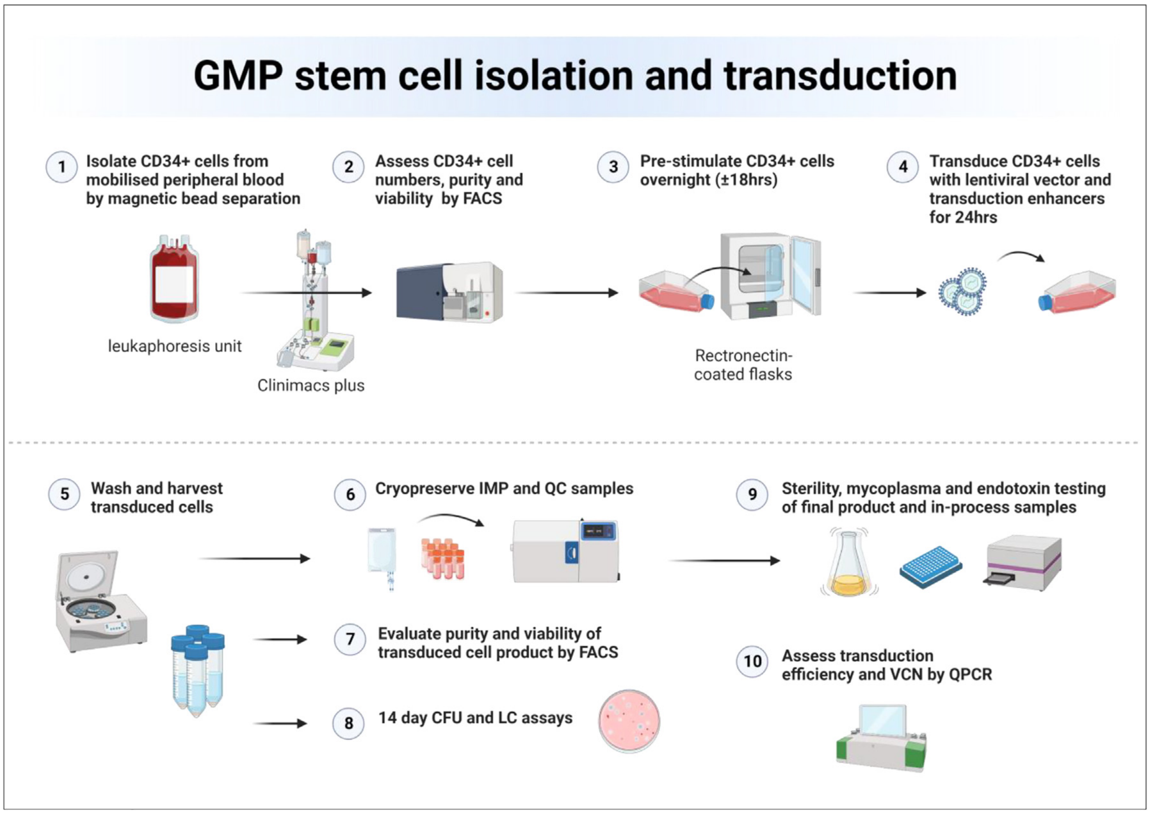

Figure 5. Overview of the GMP manufacturing process

(1) Stem cells are isolated from the leukapheresis unit by magnetic bead separation using a CliniMACS plus or prodigy instrument. (2) The number, purity, and viability of isolated $CD34^+$ cells is determined by FACS. (3) Before genetic modification, the cells undergo a pre-stimulation step in growth media + cytokines that allows more effective TD. (4) Cells are transduced with LV, which introduces correct copies of the defective gene into patient cells. (5) Following TD, cells are washed by centrifugation, cells counted, and resuspended in Cryostor at 2×10^6 cell/mL prior to being cryopreserved in a controlled rate freezer (6). The IMP to be transplanted in the patient is stored in a cryobag, while smaller vials for QC analysis are stored as cryovials. (7) Before and 24 h after cryopreservation, the viability and post-thaw recovery of the product is assessed by FACS. (8) Some of the transduced cells are seeded in colony-forming unit (CFU) and LC assays. (9) Samples of the IMP and samples taken throughout the manufacturing run are assessed for sterility, mycoplasma and endotoxin. (10) Individual colonies from the CFU assay are picked and TD efficiency assessed by determining the presence of the integrated transgene by qPCR. Pooled colonies and LCs are also assessed for VCNs.

1 and 2, respectively, and in pooled CFUs, 2.56 and 3.18. The final products were absent of mycoplasma, sterile and endotoxin levels were below 0.1 EU/mL, meeting release criteria specification (Table 3). These GMP validations allow for the first patients to be treated for MPSII in a recently opened phase 1/2 clinical trial (NCT05665166).

DISCUSSION

As an increasing number of HSCGT treatments advance toward clinical translation, it is important to address the current bottlenecks in the manufacturing of IMPs for patients. Very few GMP manufacturing sites in the UK have the capacity to isolate h $CD34^+$ cells from a patient leukapheresis and transduce them in a cleanroom environment to make an IMP. Our objective for this study was to develop, optimize, and validate a GMP cell manufacturing protocol for MPSII in a newly opened cleanroom facility in the northwest of England at NHSBT Barnsley, to expand IMP manufacturing capacity for HSCGT trials in the UK. We successfully completed this objective and compared and contrasted the protocol with existing GMP cell manufacture taking place at GOSH.

Completion of this study was necessary to satisfy Medicines and Healthcare Products Regulatory Agency regulatory requirements for IMP manufacture ahead of a phase I/II clinical trial for MPSII. No *in vivo* data demonstrating transplantation of the IMP in a humanized mouse model were required, as this procedure has been shown to work *ex vivo* by applicants on other similarly designed HSCGT clinical trials.[37] We have demonstrated effective engraftment of gene-modified HSCs in MPSII mice in our previous proof-of-concept publications.[23,24] Immunocompromised NSG mice are sensitive to busulfan conditioning, and so cannot be given full conditioning like the patients and as such are a poor model for engraftment efficiency in this case.[38]

Table 1. GMP manufacture validation runs performed at NHSBT Barnsley cleanroom

	Sample	% Viability thawed CD34+	Recovered transduced CD34+ cell × 10^6/kg[a]	% Overall recovery transduced CD34+ from cells seeded	VCN		IDS activity (μM 4-MU/4 h/mg protein) for information only		Sterility	Mycoplasma genus PCR	Endotoxin EU/mL	Meets specification
					Pooled CFU	14 day LC	Pooled CFU	14 day LC				
GMP run 1	Cells in Cryostor	96.8	127	88.8	–	–	–	–	–	–	–	yes
	Immediate post thaw	96.8	9.16	64	6.56	–	826	–	no growth	not detected	<0.1	yes
	6 weeks	96	8.09	56.6	7.55	–	1930	4085	–	–	–	yes
	12 weeks	97.1	7.82	55.2	6.27	5.09	2233	4220	–	–	–	yes
GMP run 2	Cells in Cryostor	97.1	103.67	88.2	–	–	–	–	–	–	–	yes
	Immediate post thaw	97.1	8.68	73.8	5.65	–	327	480	no growth in FP[b]	not detected	<0.1	yes
	6 weeks	94.8	7.82	70.2	5.76	–	4467	3310	–	–	–	yes
	12 weeks	98.1	9.91	84.27	6.62	6.34	3516	3840	–	–	–	yes

Two GMP validation runs were performed with the inclusion of 6- and 12-week stability studies. The percentage CD34+ cell viability was assessed by FACS, VCN by QPCR and enzyme activity by IDS activity assay. Samples were tested for sterility, mycoplasma and endotoxin contamination. Both final products met specification requirements.

[a]Estimated typical patient weight of 10 kg used in calculations.

[b]*Corynebacterium jeikium* identified in starting product only. Probable contamination at donor collection as sampling performed aseptically from unmanipulated bag.

Gene transfer into HSPCs can be challenging and historical clinical TD protocols rely on the use of high MOIs, typically combined with multiple rounds of viral vector administration and prolonged *ex vivo* culture, which is more expensive, requires more time, and may ultimately have a detrimental effect on long-term cell engraftment, favoring the expansion of more committed HPCs at the cost of repopulating HSCs.[39,40] In a recent phase 1/2 HSCGT clinical trials for MPSIIIA, the TEs LentiBOOST and protamine sulfate, were successfully used to significantly improve TD and therefore reduce vector amounts and costs for IMP manufacture (NCT04201405, unpublished data).[28] Here, we demonstrated the effectiveness of the same TE combination to significantly improve TD of hCD34+ stem cells with IDS.ApoEII LV for the treatment of MPSII. Overall, in our small-scale TD optimization experiments, we observed an approximate 2.5-fold increase in VCN in LC samples and 2.9-fold increase for pooled CFU at the 12.5, 25, and 50 MOI vector concentrations with TEs. IDS activity similarly increased in cells transduced with TEs. The inclusion of IL-3 in the growth media further amplified the effects of TEs. However, with the highest vector concentration tested, MOI 100, we observed significant toxicity in the CFU assay, most likely due to massive overexpression of the therapeutic gene in those cells to toxic levels. CFU counts were significantly down at MOI 100 + TE, with the remaining surviving colonies being those present with a lower VCN. Potential improvements to TD with IDS.ApoEII LV may be possible by further optimization of TE type and concentrations; however, this was outside the scope of the project.

When scaling up the manufacturing process to transducing an at-scale amount of cells in a T75 flask, comparable VCNs were achieved at LV concentrations of MOI 60 without TEs and MOI 12.5 with TEs, indicating that the enhancers can allow a significant decrease in vector amounts by almost 5-fold, while achieving equivalent efficacy in terms of VCN and IDS activity. For these initial research and development (R&D) studies, we were using X-VIVO-15 (Lonza) media and cytokines Flt-3 ligand, SCF, TPO, and IL-3 (Peprotech) in the TD media. Several current HSCGT clinical trial manufacturing protocols have moved to using GMP SCGM due to its ability to support greater expansion in HSPCprim percentage compared with X-VIVO-15 and HSC brew.[28,41] For our GMP process at the NHSBT Barnsley facility, our initial aim was to use SGCM media; however, this study was being conducted during the coronavirus disease 2019 pandemic and unfortunately SCGM was on back order for >9 months with supply being prioritized for active clinical trials only. For this reason, we opted to continue process development with GMP X-VIVO-15 media, which demonstrates comparable results with SCGM in terms of total cell counts and HSPC maintenance and expansion.[28] Many BMRs have flexibility, allowing for the use of flasks or culture bags depending on consumable availability. In our initial R&D scale up work at the NHSBT Barnsley site, we compared culturing CD34+ cells in flasks vs. a Vuelife bag; however, we identified bacterial contamination from *Staphylococcus haemolyticus* in the cells cultured in the Vuelife bag and as such the data were omitted from this manuscript. Bacterial contamination was most likely a consequence of our limited experience at the time using this system.

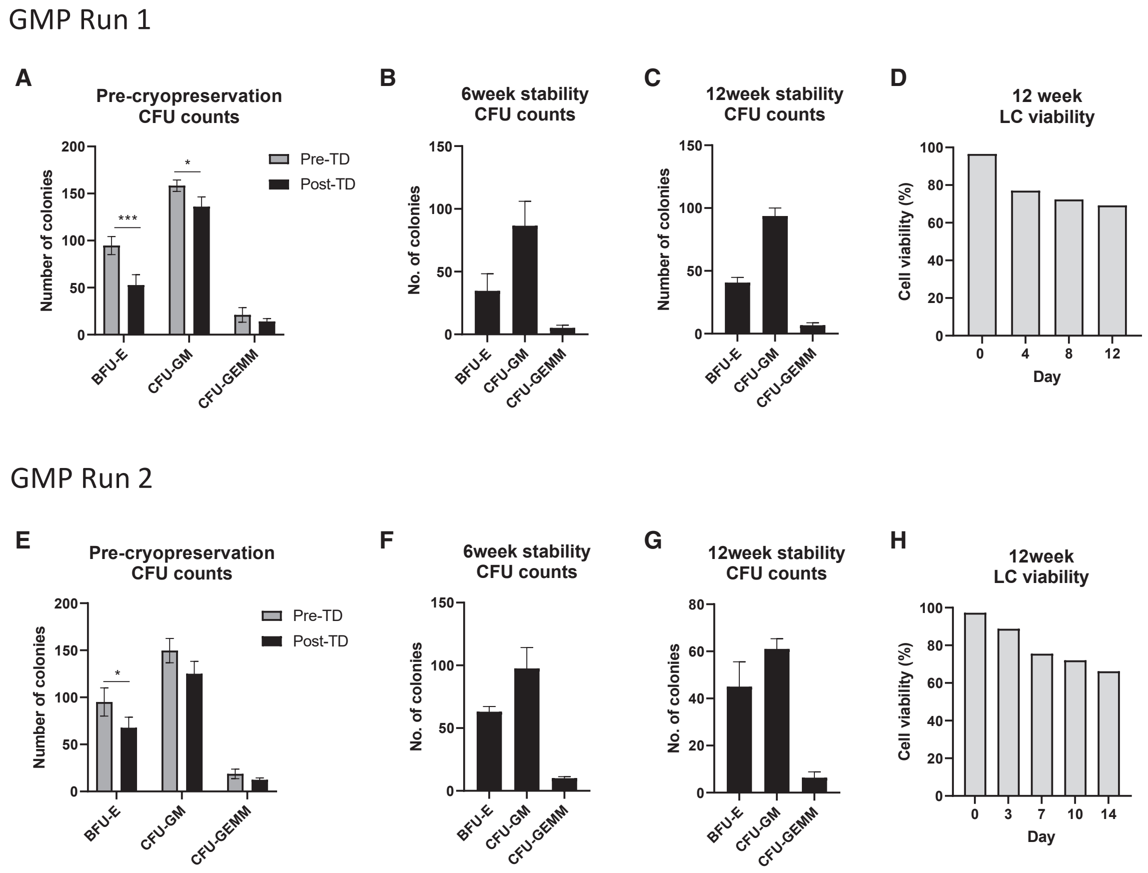

Figure 6. CFU and LC assays from Barnsley GMP runs 1 and 2

CFU colony counts from run 1 GMP product pre-cryopreservation (A), and following 6 and 12 weeks cryopreservation (B and C). (D) GMP run 1: 12-week stability and 14-day LC cell viability. CFU colony counts from run 2 GMP product pre-cryopreservation (E), and following 6 and 12 weeks cryopreservation (F and G). (H) GMP run 1 12 week stability 14 day LC cell viability. Data are mean ± SEM. One-way ANOVA. ***$p < 0.001$. *$p < 0.05$.

The use of culture bags should in theory decrease the risk of contamination due to the reduced number of open manipulations.

Moving to cleanroom GMP validation runs conducted at NHSBT, we took forward our TD protocol utilizing X-VIVO-15-based TD media, including IL-3 and using retronectin coated T175 flasks, similar to other HSCGT methods such as for Artemis-deficient SCID and ADA-SCID,[31,42] and transducing cells with GMP IDS.ApoEII LV at an MOI of 25 with TEs. After two manufacturing runs, we achieved consistently high VCNs (approximately 5–7 copies) in the 14-day CFU assays and LCs seeded with cryopreserved product immediately post-thaw and following 6- and 12-week cryopreservation, indicating the product is stable for at least 12 weeks. Both GMP products met specification with CD34$^+$ cell viability of 94.8% or above and high recovery rates after thawing. We compared the manufacturing process developed at NHSBT, with GMP validations performed at GOSH, using a protocol that uses SCGM media without IL-3 and the same batch of GMP vector. The GOSH TD step also uses approximately one-third less vector at equivalent MOIs, due to the differences in LV titer determination. Under these conditions, greater vector concentrations were required to achieve similar VCNs (Tables 3, S2, and S3). An MOI of 100 + TEs resulted in VCNs in the 2.3–3.3 range from two validation runs and was not toxic at this concentration, unlike the small-scale TD studies performed at Manchester, where IL-3 was included (Figure 2).

There has been much debate about inclusion of IL-3 in the cytokine cocktail, with some current HSCGT protocols include IL-3 in TD media, where others exclude the cytokine.[35,43,44] It was evident from our studies that including IL-3 in addition to the standard SCF, FLT-3, and TPO cytokines increased VCN and IDS enzyme activity when evaluated after short-term CFU and LCs, in agreement with findings from other studies evaluating LV-EFS-ADA TD of CD34$^+$ cells for ADA deficiency.[45] Carbonaro et al.[45] suggested in their work that the higher VCN observed in short-term bulk culture and CFUs was a consequence of a subset of progenitors receiving increased vector copies, rather than significant TD of additional cells. In our hands, with the addition of IL-3 to the media and in combination with TEs for the TD step, we detected vector copies in 94%–100% of transduced CD34$^+$ cells following large-scale TDs with an IDS.ApoEII LV

Table 2. Comparison of the Barnsley and GOSH GMP manufacturing protocols

GMP facility	CD34 cell selection	GMP TD media	Culture vessel	IDS.ApoEII LV titer
NHSBT, Barnsley	Clinimacs Plus	X-VIVO15, 1% HAS, 300 ng/mL SCF, 300 ng/mL FLT-3, 100 ng/mL TPO, 20 ng/mL IL3	flask	1.0×10^9 TU/mL
GOSH, London	Clinimacs Prodigy	SCGM, 1% HAS, 300 ng/mL SCF, 300 ng/mL FLT-3, 100 ng/mL TPO	bag or flask	1.4×10^9 TU/mL

concentration of MOI 25, with a similar distribution of vector insert in those cells (Table S5). Exclusion of IL-3 resulted in overall lower vector copies and to compensate, higher vector concentrations were required. For the imminent phase 1/2 clinical trial of HSCGT in MPSII patients using IDS.ApoEII vector, IL-3 will be excluded from the GMP manufacturing protocol to maximize the number of early progenitors for engraftment; however, TEs will be implemented to decrease the concentration of LV required.

In summary, we have designed, optimized and validated a new GMP stem cell TD protocol for MPSII utilizing TEs in preparation for future HSCGT clinical trials in the UK. Future development work will look toward fully enclosing the system and to adopt a semi-automated manufacturing process.

MATERIALS AND METHODS

Vector manufacture and titer

Sufficient quantities (>4 mL total) of concentrated, VSV-G pseudotyped research-grade CD11b.IDS.ApoEII.WPRE LV (IDS.ApoEII LV),[24] for both small and at-scale TD validations, were produced via the following method. HEK 293T cells of low passage were seeded in 65 × 15-cm tissue culture plates (Corning) in DMEM/10% fetal calf serum (FCS)/2 mmol/l L-glutamine (Lonza) and cultured overnight at 37°C/5% CO_2 until 70%–80% confluent. Confluent HEK 293T cells were transiently transfected with third-generation plasmids composed of pCCLsin.hCD11b.IDS.ApoEII.WPRE, pMDG, pMDLgpRRE, and pRSVREV in a 2:1:1:2 ratio using a total of 12 μg plasmid DNA per dish. Plasmid DNA sufficient for three plates was added to 3 mL TD media containing 150 mM NaCl and 0.5 mM polyethyenimine (molecular weight = 40 kDa) (Polysciences). We added 1 mL of the TD media to each plate in a dropwise fashion and the plates were incubated overnight (approximately 17 h) at 37°C/5% CO_2. The following morning, media were replaced and supernatant harvested at 24 and 48 h. Any detached cells were removed by centrifugation at 200×*g* for 5 min at 4°C, prior to filtration through a 0.45-μm low protein-binding filter (Nalgene). Supernatent harvests were pooled and LV particles were concentrated by centrifugation at 21,191×*g* for 150 min at 4°C and then resuspended in formulation buffer (tissue culture grade PBS, 1 mg/mL human serum albumin, 5 μg/mL protamine sulfate, 40 mg/mL lactose, pH 7.2), aliquoted and stored at −80°C until ready for use.

Clinical-grade IDS.ApoEII LV was produced to Good Manufacturing Practices (GMPs) at Indiana vector production Facility (concentrated from a 60-L batch) and QC testing was performed prior to final vector release.

The titer of LVs was determined by both qPCR and droplet digital PCR (ddPCR)-based methods. We cultured 2×10^5 HCT116 cells (ATCC CCL-247) in McCoy's 5a Medium (Lonza)/10% FCS/2 mmol/L L-glutamine and transduced with 10-fold serial dilutions of concentrated LV starting at 1 in 100. Four days later, genomic DNA was extracted from the cells using the GenElute Mammalian Genomic DNA Miniprep Kit (Sigma-Aldrich) and analyzed by quantitative PCR and ddPCR to determine the number of integrated lentiviral genomes per cell. HIV forward primer (100 μM) (5′-TCTCGACGCAGGACTCG-3′), HIV reverse primer (100 μM) (5′-TACTGACGCTCTCGCACC-3′), SDC4 forward primer (100 μM) (5′-CAGGGTCTGGGAGCCAAGT-3′) and SDC4 reverse primer (100 μM) (5′-GCACAGTGCTGGACATTGACA-3′) were purchased from Integrated DNA Technologies as were the HIV probe (100 μM) (5′-ATCTCTCTCCTTCTAGCCTC-3′ FAM/ZEN-IOWA) and SDC4 probe (100 μM) (5′-CCCACCGAACCCAAGAAACTAGAGGAGAAT-3′ HEX/ZEN-IOWA). The infectious titer was calculated as the number of cells at TD multiplied by the number of lentiviral copies per cell divided by the volume of LV added. The titer of LVs at GOSH was performed using a similar HIV PSI ddPCR approach but using an HT29 cell line for TD.

TD optimization at small scale

Cryopreserved CD34+ cells (mobilized peripheral blood mononuclear cells derived) were thawed by rapidly incubating at 37°C and then adding 10× volume of IMDM (Lonza) + 5% FBS (Sigma), with a 10-min recovery time at room temperature (RT). Cells were centrifuged for 200×*g* for 10 min at RT prior to being resuspended in 1mL growth media (x-vivo 15 [Lonza] + 1% HSA [Bio Products Laboratory Ltd], 100 ng/mL TPO, 300 ng/mL SCF, 300 ng/mL Flt3-L, 20 ng/mL IL-3 [cytokines from Peprotech]). The number and viability of cells was assessed by trypan blue staining. In a 48-well plate, CD34+ cells were seeded at a density of 5×10^5–1×10^6 cells/mL in growth media (500 μL) and prestimulated for 18 ± 2 h at 37°C/5%CO_2. The number of cells in a representative well were counted by hemacytometer and the volume of LV to add calculated by the following equation: LV (mL) = (MOI × cell number)/LV titer (TU/mL). Cells were transferred to 1.5-mL Eppendorf's and centrifuged at 300×*g* for 5 min at RT then resuspended in 450 μL growth media. The desired amount of LV plus TEs were added (LentiBOOST (1 mg/mL, Sirion Biotech) and protamine sulfate [4 μg/mL, Wockhardt UK]). The total volume made up to 500 μL with growth media and cells transferred back to the 48-well plate. Cells were transduced for 24 ± 2 h at 37°C/5% CO_2 and washed by centrifugation and resuspending in fresh growth media. Cells were then seeded in the CFU assay.

Table 3. GMP validation runs 1 and 2 performed at GOSH cleanroom facility

		Condition						Condition			
GMP run1		DP	UNT	Acceptance criteria	Pass/ fail	GMP run2		DP	UNT	Acceptance criteria	Pass/ fail
Cell viability	day 1	N/A	91.09%	≥70% viable cells	pass	cell viability	day 1	98.27%	98.27%	≥70% viable cells	pass
	day 3	91.79%	92.27%				day 3	91.63%	N/A		
VCN	liquid	3.3	0	≥0.5	pass	VCN	liquid	2.25	0	≥0.5	pass
	CFUs	2.56	0	for information only	N/A		CFUs	3.175	0.0085	for information only	N/A
CFU content		508 CFUs/1,000 cells	596 CFUs/1000 cells	≥4 CFUs/1,000 cells plated	pass	CFU content		398 CFUs/1000 cells	338 CFUs/1000 cells	≥4 CFUs/1000 cells plated	Pass
		achieved ≥50% of UNT value	N/A for UNT	≥50% of the UNT control value	pass			Achieved ≥50% of UNT value	N/A for UNT	≥50% of the UNT control value	Pass
		mean 41.73 BFU-E and 51.97 CFU-GM counted	mean 30.2 BFU-E and 66.44 CFU-GM counted	≥1 BFU-E and ≥1 CFU-GM counted	pass			mean 45.73 BFU-E and 50.25 CFU-GM counted	mean 42.01 BFU-E and 51.48 CFU-GM counted	≥1 BFU-E and ≥1 CFU-GM counted	pass
CD34 expression		99.80%	99.50%	for information only	N/A	CD34 expression		97.00%	99.60%	for information only	N/A
Mycoplasma		not detected	N/A	not detected	pass	mycoplasma		Not Detected	N/A	not detected	pass
Sterility		no growth	N/A	no growth	pass	sterility		No Growth	N/A	no growth	pass
Endotoxin		<0.100 EU/mL	N/A	≤5 EU/mL	pass	endotoxin		<0.100 EU/mL	N/A	≤5 EU/mL	pass
						IDS activity	liquid	64.4	8.82	for information only	N/A
							CFUs	1511.9	33.7		

DP, transduced cell product; UNT, un-transduced cells.

CFU assay and LC

Vials of 4 mL methocult (H4034 Stem Cell Technologies) were thawed overnight in the fridge or on the bench at RT prior to use. One thousand cells from each treatment group were added to a total of 400 μL IMDM (Lonza) and then added to 4 mL methocult using a P1000 pipette with gentle stirring. The methocult media was then divided into 3 × 3.5 cm dishes using a 16G blunt-ended needle and a 5-mL syringe as per manufacturer's instructions. Dishes were incubated in a humid environment at 37°C/5%CO_2 for 14 days. Colonies were identified by visual inspection using a light microscope following the manufacturer's guidelines. Eighteen individual BFU-E and CFU-GM colonies were picked under the microscope using a P2 pipette. Remaining colonies were pooled across the three dishes and divided for VCN and enzyme assay analysis. For LC samples, transduced and non-transduced $CD34^+$ cells were seeded at 0.5 × 10^6 cells/mL in a six-well plate in growth media (x-vivo 15 + 1% HSA, 100 ng/mL TPO, 300 ng/mL SCF, 300 ng/mL Flt3-L, 20 ng/mL IL-3) for 14 days with media change twice per week. For media changes, cells were transferred to a 1.5-mL tube and spun at 200×*g* for 5 min at RT, with 100 μL of growth media added to wells to cover any adherent cells. Cell pellets were resuspended in growth media, counted with trypan blue to assess viability and re-seeded at 0.5 × 10^6 cells/mL per well.

VCN and TD efficiency assessment

The number of integrated vector copies in transduced cells were assessed by qPCR using primer and probe sets described above. For pooled CFU and LCs gDNA was extracted and eluted in 200 μL elution buffer as per kit instructions (Sigma GenElute Mammalian Genomic DNA Miniprep Kit). For individual colonies, gDNA was eluted in 50 μL. TD efficiency was determined by assessing VCN in individual colonies by qPCR (36 per group). Positive colonies were defined as having more than 0.3 copies on integrated insert. The TD efficiency was calculated by dividing the number of positive colonies by total colonies × 100.

IDS enzyme activity assay

IDS enzyme activity was measured in a two-step protocol using the fluorescent substrate MU-αIdoA-2S (Carbosynth) and Aldurazyme (Genzyme) as previously described.[46] Starting material was standardized to 5–10 μg total protein for $CD34^+$cells using a BCA assay (Thermo Fisher Scientific). Fluorescence was measured using the BioTek Synergy HT plate reader (excitation, 360 nm; emission, 460 nm).

Flow cytometry

Flow cytometry was performed using a Beckmann Coulter Gallios FACS instrument. CD34 and CD45 fluorescent antibodies and a 7AAD live dead stain (Beckmann Coulter) were used to determine CD34 stem cell purity and viability. The method in brief used two Trucount tubes (Becton Dickinson) for each sample to be tested in duplicate. Ten microliters of CD34-PE, CD45-FITC, and 7-AAD were added to the tubes followed by 50 μL of diluted sample. The cells and antibodies were mixed by shaking tubes within the rack, followed

by an incubation in the dark for 15 min at 18°C–25°C. One milliliter of diluted lysing solution was added to all tubes and then mixed with an incubation in the dark at 18°C–25°C for a further 5–10 min before being run of the flow cytometer.

Pilot at-scale manufacturing validations

An mPBSC leukapheresis unit was purchased from the AN and CD34$^+$ cells isolated by magnetic bead separation using a clinimacs plus instrument and CD34$^+$ cell separation kit (Miltenyi Biotech). A T175 flask was pre-coated with 16 mL of 20 μg/mL retronectin solution (Takara Bio) overnight at 4°C. The flask was then incubated in blocking solution (2% HAS [Bio Products Laboratory Ltd] in PBS) for 30 min at RT and then washed with PBS. CD34$^+$ stem cells were prestimulated overnight for 18 ± 2 h in a rectronectin-coated T175 flask or VueLife culture bag in culture media containing IL-3 (X-VIVO 15 [Lonza], 100 ng/mL TPO, 300 ng/mL SCF, 300 ng/mL FLT3, 20 ng/mL IL-3 [cytokines from Peprotech], 1% HAS [Bio Products Laboratory Ltd]) or in a VueLife culture bag absent of IL-3 in culture media (X-VIVO 15, 100 ng/mL TPO, 300 ng/mL SCF, 300 ng/mL FLT3, 1% HAS). CD34$^+$ cells were transduced with research-grade IDS.ApoEII LV with TEs LentiBOOST (1 mg/mL, Sirion Biotech) and protamine sulfate (4 μg/mL, Wockhardt UK) for 24 ± 2 h. CD34$^+$ cell viability and purity were assessed by FACS and cells seeded in CFU and LC assays.

GMP manufacturing validations

Large-scale GMP manufacturing runs performed at NHSBT Barnsley cleanroom facility. All reagents and consumables used were GMP compliant. Leukapheresis units were purchased from the AN and CD34$^+$ cells isolated by magnetic bead separation using a clinimacs plus instrument and CD34$^+$ cell separation kit (Miltenyi Biotech). CD34$^+$ stem cells were prestimulated overnight for ±18 h in culture media (lonza x-vivo 15, IL-3, TPO, SCF, FLT3, and HSA) in rectronectin-coated T175 flasks. CD34$^+$ cells were transduced with GMP IDS.ApoEII LV with TEs lentiboost (1 mg/mL, Sirion Biotech) and protamine sulfate (4 μg/mL, Wockhardt UK) for ±24 h. The next day, cells were washed twice (0.9% normal saline, 1% HAS) and cryopreserved at 2×10^6 cell/mL in CryoStor CS5 containing 5% DMSO in KryoSure 20-F cryobag (Saint-Gobain) using a controlled rate freezer (Planer Kryo 560). The cryopreserved product, in volumes of 20 ± 2.5 mL, is then stored in the vapor phase of LN_2 (VPLN) at a temperature of −130°C or less. Cryopreservation protocol (Kryo 560-16 controlled rate freezer): Hold at 4°C for 5 min, first ramp cools at a rate of −1°C/min to −30°C, second ramp cools at a rate of −2°C/min to −60°C, and ramp three cools at a rate of −20°C/min to −180°C. Hold at −180°C until unloaded into VPLN upon completion of program. Cells were transferred immediately to VPLN storage.

QC testing

Sterility testing was performed to European Pharmacopeia by the Scottish National Blood Transfusion Service (Jack Copeland Center). Sampling time points throughout the manufacturing process and volumes taken are detailed in Table S6. Endotoxin testing on 100 μL of cryopreserved final product was performed by NHSBT (Clinical Biotechnology Center). *Mycoplasma* PCR was performed on spent TD media from day 4 of the manufacturing run by Micropathology (University of Warwick Science Park).

Large-scale GMP manufacturing runs performed at GOSH cleanroom facility—StemExpress (USA) G-CSF mobilized leukapheresis units were purchased from Caltag and CD34$^+$ cells isolated by magnetic bead separation using a clinimacs prodigy instrument and CD34$^+$ cell separation kit (Miltenyi Biotech). CD34$^+$ stem cells were prestimulated overnight for 20 ± 6 h in culture media (GMP SCGM [Cellgenix], 100 ng/mL TPO, 300 ng/mL SCF, 300 ng/mL Flt3-L [cytokines from Cellgenix], 1% HAS [Bio Products Laboratory Ltd]) in Vuelife cell culture bags (Saint Gobain) at 37°C/5% CO_2. CD34$^+$ cells were transduced at MOI 100 with GMP IDS.ApoEII LV for 18 ± 6 h, washed and cryopreserved at 17.5×10^6 cells/mL or less.

Cryopreservation protocol

The start temperature was 4°C; the first ramp cools at a rate of −1°C/min to −20°C, hold for 5 min at −20°C, and the second ramp cools at a rate of −2°C/min to −80°C, hold for 10 min. Specification for batch release testing of the cryopreserved product is described in Table S1. Barnsley MOI vs. GOSH MOI vector concentrations used are compared in Table S4.

DATA AND CODE AVAILABILITY

Raw data were generated at University of Manchester, NHSBT Barnsley, and GOSH, London. Derived data supporting the findings of this study are available from the corresponding author (B.W.B.) on request if authorized by our study sponsor.

SUPPLEMENTAL INFORMATION

Supplemental information can be found online at https://doi.org/10.1016/j.omtm.2024.101271.

ACKNOWLEDGMENTS

We would like to thank the Innovate Manchester Advanced Therapy Center Hub (iMATCH) led by Fiona Thistlethwaite and Innovate UK (UKRI) for funding a large proportion of the work. We would also like to thank Jon Smythe and the GMP team at NHSBT Barnsley, led by Victoria Day, for the cleanroom collaboration and all the team at Great Ormond Street Hospital (GOSH) for their GMP validation work. Work done at GOSH was funded by Avrobio and supported by the NIHR Great Ormond Street Biomedical Research Center. Finally, we would like to acknowledge Miltenyi for their assistance in setting up the CliniMACS instrument and the training provided and the Anthony Nolan Trust for the supply of high quality leukapheresis units.

AUTHOR CONTRIBUTIONS

S.E. and B.W.B. wrote the manuscript. S.E. and B.W.B. secured the funding for the study. S.E. designed and led the studies performed at UoM and NHSBT. K.B. led studies performed at GOSH. V.D. and J.S. oversaw studies ran in the cleanroom facilities at NHSBT and inputed into experimental design and assay development for

release criteria. S.E., Y.L., S.K., and L.H. performed cleanroom studies at NHSBT. K.B., F.J.R.R., and J.B. performed cleanroom studies at GOSH. C.B. and A.T. oversaw cleanroom studies performed at GOSH. L.B., R.H., and S.J. provided assistance with validation study design to meet regulatory standards.

DECLARATION OF INTERESTS

B.W.B. holds a patent for the use of the IDS.ApoEII in the treatment of MPSII and is the recipient of a clinical trial grant from AVROBIO for the treatment of MPSII with LV.IDS.ApoEII.

REFERENCES

1. Fox, T.A., and Booth, C. (2021). Gene therapy for primary immunodeficiencies. Br. J. Haematol. *193*, 1044–1059. https://doi.org/10.1111/bjh.17269.
2. Poletto, E., Silva, A.O., Weinlich, R., Martin, P.K.M., Torres, D.C., Giugliani, R., and Baldo, G. (2023). Ex vivo gene therapy for lysosomal storage disorders: future perspectives. Expert Opin. Biol. Ther. *23*, 353–364. https://doi.org/10.1080/14712598.2023.2192348.
3. Segura, E.E.R., Ayoub, P.G., Hart, K.L., and Kohn, D.B. (2023). Gene Therapy for β-Hemoglobinopathies: From Discovery to Clinical Trials. Viruses *15*, 713. https://doi.org/10.3390/v15030713.
4. Fumagalli, F., Calbi, V., Natali Sora, M.G., Sessa, M., Baldoli, C., Rancoita, P.M.V., Ciotti, F., Sarzana, M., Fraschini, M., Zambon, A.A., et al. (2022). Lentiviral haematopoietic stem-cell gene therapy for early-onset metachromatic leukodystrophy: long-term results from a non-randomised, open-label, phase 1/2 trial and expanded access. Lancet *399*, 372–383. https://doi.org/10.1016/S0140-6736(21)02017-1.
5. Kohn, D.B., Booth, C., Kang, E.M., Pai, S.-Y., Shaw, K.L., Santilli, G., Armant, M., Buckland, K.F., Choi, U., De Ravin, S.S., et al. (2020). Lentiviral gene therapy for X-linked chronic granulomatous disease. Nat. Med. *26*, 200–206. https://doi.org/10.1038/s41591-019-0735-5.
6. Kinsella, J.L., Wynn, R.F., Bigger, B., Thrasher, A.J., Booth, C., Buckland, K., Izotova, N., Rust, S., Weisberg, D., Church, H.J., et al. (2020). Ex-Vivo Autologous Stem Cell Gene Therapy Clinical Trial for Mucopolysaccharidosis Type IIIA: Trial in Progress - NCT04201405. Blood *136*, 15–16. https://doi.org/10.1182/blood-2020-141762.
7. Biffi, A. (2018). Gene Therapy as a Curative Option for beta-Thalassemia. N. Engl. J. Med. *378*, 1551–1552. https://doi.org/10.1056/NEJMe1802169.
8. Eichler, F., Duncan, C., Musolino, P.L., Orchard, P.J., De Oliveira, S., Thrasher, A.J., Armant, M., Dansereau, C., Lund, T.C., Miller, W.P., et al. (2017). Hematopoietic Stem-Cell Gene Therapy for Cerebral Adrenoleukodystrophy. N. Engl. J. Med. *377*, 1630–1638. https://doi.org/10.1056/NEJMoa1700554.
9. De Ravin, S.S., Wu, X., Moir, S., Anaya-O'Brien, S., Kwatemaa, N., Littel, P., Theobald, N., Choi, U., Su, L., Marquesen, M., et al. (2016). Lentiviral hematopoietic stem cell gene therapy for X-linked severe combined immunodeficiency. Sci. Transl. Med. *8*, 335ra57. https://doi.org/10.1126/scitranslmed.aad8856.
10. Gaspar, H.B., Cooray, S., Gilmour, K.C., Parsley, K.L., Zhang, F., Adams, S., Bjorkegren, E., Bayford, J., Brown, L., Davies, E.G., et al. (2011). Hematopoietic stem cell gene therapy for adenosine deaminase-deficient severe combined immunodeficiency leads to long-term immunological recovery and metabolic correction. Sci. Transl. Med. *3*, 97ra80. https://doi.org/10.1126/scitranslmed.3002716.
11. Harrison, C. (2019). First gene therapy for beta-thalassemia approved. Nat. Biotechnol. *37*, 1102–1103. https://doi.org/10.1038/d41587-019-00026-3.
12. Aiuti, A., Roncarolo, M.G., and Naldini, L. (2017). Gene therapy for ADA-SCID, the first marketing approval of an ex vivo gene therapy in Europe: paving the road for the next generation of advanced therapy medicinal products. EMBO Mol. Med. *9*, 737–740. https://doi.org/10.15252/emmm.201707573.
13. European Medicines Agency (2021). Libmeldy approved for use in the european union. https://www.ema.europa.eu/en/medicines/human/EPAR/libmeldy.
14. Bigger, B.W., and Wynn, R.F. (2014). Novel approaches and mechanisms in hematopoietic stem cell gene therapy. Discov. Med. *17*, 207–215.
15. Karpova, D., Rettig, M.P., and DiPersio, J.F. (2019). Mobilized peripheral blood: an updated perspective. F1000Res. *8*. F1000 Faculty Rev-2125. https://doi.org/10.12688/f1000research.21129.1.
16. Staal, F.J.T., Aiuti, A., and Cavazzana, M. (2019). Autologous Stem-Cell-Based Gene Therapy for Inherited Disorders: State of the Art and Perspectives. Front. Pediatr. *7*, 443. https://doi.org/10.3389/fped.2019.00443.
17. Bernardo, M.E., and Aiuti, A. (2016). The Role of Conditioning in Hematopoietic Stem-Cell Gene Therapy. Hum. Gene Ther. *27*, 741–748. https://doi.org/10.1089/hum.2016.103.
18. Sagoo, P., and Gaspar, H.B. (2023). The transformative potential of HSC gene therapy as a genetic medicine. Gene Ther. *30*, 197–215. https://doi.org/10.1038/s41434-021-00261-x.
19. Wilkinson, F.L., Holley, R.J., Langford-Smith, K.J., Badrinath, S., Liao, A., Langford-Smith, A., Cooper, J.D., Jones, S.A., Wraith, J.E., Wynn, R.F., et al. (2012). Neuropathology in mouse models of mucopolysaccharidosis type I, IIIA and IIIB. PLoS One *7*, e35787. https://doi.org/10.1371/journal.pone.0035787.
20. Ellison, S., Parker, H., and Bigger, B. (2023). Advances in therapies for neurological lysosomal storage disorders. J. Inherit. Metab. Dis. *46*, 874–905. https://doi.org/10.1002/jimd.12615.
21. Stapleton, M., Kubaski, F., Mason, R.W., Yabe, H., Suzuki, Y., Orii, K.E., Orii, T., and Tomatsu, S. (2017). Presentation and Treatments for Mucopolysaccharidosis Type II (MPS II; Hunter Syndrome). Expert Opin. Orphan Drugs *5*, 295–307. https://doi.org/10.1080/21678707.2017.1296761.
22. Wraith, J.E., Scarpa, M., Beck, M., Bodamer, O.A., De Meirleir, L., Guffon, N., Meldgaard Lund, A., Malm, G., Van der Ploeg, A.T., and Zeman, J. (2008). Mucopolysaccharidosis type II (Hunter syndrome): a clinical review and recommendations for treatment in the era of enzyme replacement therapy. Eur. J. Pediatr. *167*, 267–277. https://doi.org/10.1007/s00431-007-0635-4.
23. Ellison, S., Liao, A., Gleitz, H.F.E., Parker, H., Booth, L., Robinson, J., Wood, S., Taylor, J., Holley, R., and Bigger, B.W. (2023). Sustained long-term disease correction in a murine model of MPSII following stem cell gene therapy. Mol. Ther. Methods Clin. Dev. *31*, 101127. https://doi.org/10.1016/j.omtm.2023.101127.
24. Gleitz, H.F., Liao, A.Y., Cook, J.R., Rowlston, S.F., Forte, G.M., D'Souza, Z., O'Leary, C., Holley, R.J., and Bigger, B.W. (2018). Brain-targeted stem cell gene therapy corrects mucopolysaccharidosis type II via multiple mechanisms. EMBO Mol. Med. *10*, e8730. https://doi.org/10.15252/emmm.201708730.
25. Mandolfo, O., Liao, A., Singh, E., O'Leary, C., Holley, R.J., and Bigger, B.W. (2024). Establishment of the Effectiveness of Early Versus Late Stem Cell Gene Therapy in Mucopolysaccharidosis II for Treating Central Versus Peripheral Disease. Hum. Gene Ther. *35*, 243–255. https://doi.org/10.1089/hum.2023.002.
26. Bischof, D., and Cornetta, K. (2022). Indiana University Vector Production Facility (IUVPF). In Cell Therapy: cGMP Facilities and Manufacturing, A.P. Gee, ed. (Springer International Publishing), pp. 339–349. https://doi.org/10.1007/978-3-030-75537-9_20.
27. Panch, S.R., Reddy, O.L., Li, K., Bikkani, T., Rao, A., Yarlagadda, S., Highfill, S., Fowler, D., Childs, R.W., Battiwalla, M., et al. (2019). Robust Selections of Various Hematopoietic Cell Fractions on the CliniMACS Plus Instrument. Clin. Hematol. Int. *1*, 161–167. https://doi.org/10.2991/chi.d.190529.001.
28. Schott, J.W., León-Rico, D., Ferreira, C.B., Buckland, K.F., Santilli, G., Armant, M.A., Schambach, A., Cavazza, A., and Thrasher, A.J. (2019). Enhancing Lentiviral and Alpharetroviral Transduction of Human Hematopoietic Stem Cells for Clinical Application. Mol. Ther. Methods Clin. Dev. *14*, 134–147. https://doi.org/10.1016/j.omtm.2019.05.015.
29. Jang, Y., Kim, Y.S., Wielgosz, M.M., Ferrara, F., Ma, Z., Condori, J., Palmer, L.E., Zhao, X., Kang, G., Rawlings, D.J., et al. (2020). Optimizing lentiviral vector transduction of hematopoietic stem cells for gene therapy. Gene Ther. *27*, 545–556. https://doi.org/10.1038/s41434-020-0150-z.
30. Gaspar, H.B., Bjorkegren, E., Parsley, K., Gilmour, K.C., King, D., Sinclair, J., Zhang, F., Giannakopoulos, A., Adams, S., Fairbanks, L.D., et al. (2006). Successful reconstitution of immunity in ADA-SCID by stem cell gene therapy following cessation of PEG-ADA and use of mild preconditioning. Mol. Ther. *14*, 505–513. https://doi.org/10.1016/j.ymthe.2006.06.007.

31. Kohn, D.B., Booth, C., Shaw, K.L., Xu-Bayford, J., Garabedian, E., Trevisan, V., Carbonaro-Sarracino, D.A., Soni, K., Terrazas, D., Snell, K., et al. (2021). Autologous Ex Vivo Lentiviral Gene Therapy for Adenosine Deaminase Deficiency. N. Engl. J. Med. *384*, 2002–2013. https://doi.org/10.1056/NEJMoa2027675.

32. Wang, X., and Rivière, I. (2017). Genetic Engineering and Manufacturing of Hematopoietic Stem Cells. Mol. Ther. Methods Clin. Dev. *5*, 96–105. https://doi.org/10.1016/j.omtm.2017.03.003.

33. Schumm, M., Lang, P., Taylor, G., Kuçi, S., Klingebiel, T., Bühring, H.J., Geiselhart, A., Niethammer, D., and Handgretinger, R. (1999). Isolation of highly purified autologous and allogeneic peripheral CD34+ cells using the CliniMACS device. J. Hematother. *8*, 209–218. https://doi.org/10.1089/106161299320488.

34. Sergijenko, A., Langford-Smith, A., Liao, A.Y., Pickford, C.E., McDermott, J., Nowinski, G., Langford-Smith, K.J., Merry, C.L.R., Jones, S.A., Wraith, J.E., et al. (2013). Myeloid/Microglial driven autologous hematopoietic stem cell gene therapy corrects a neuronopathic lysosomal disease. Mol. Ther. *21*, 1938–1949. https://doi.org/10.1038/mt.2013.141.

35. Schejtman, A., Vetharoy, W., Choi, U., Rivat, C., Theobald, N., Piras, G., Leon-Rico, D., Buckland, K., Armenteros-Monterroso, E., Benedetti, S., et al. (2021). Preclinical Optimization and Safety Studies of a New Lentiviral Gene Therapy for p47(phox)-Deficient Chronic Granulomatous Disease. Hum. Gene Ther. *32*, 949–958. https://doi.org/10.1089/hum.2020.276.

36. Ivanovic, Z. (2004). Interleukin-3 and ex vivo maintenance of hematopoietic stem cells: facts and controversies. Eur. Cytokine Netw. *15*, 6–13.

37. Jones, S., Kinsella, J., Holley, R., Potter, J., Booth, C., Buckland, K., Rust, S., Church, H.J., Brammeier, K.L., Lee, H., et al. (2023). Sustained biochemical engraftment and early clinical outcomes following autologous stem cell gene therapy for mucopolysaccharidosis type IIIA. Mol. Genet. Metabol. *138*, 107171. https://doi.org/10.1016/j.ymgme.2022.107171.

38. Ellison, S.M., Liao, A., Wood, S., Taylor, J., Youshani, A.S., Rowlston, S., Parker, H., Armant, M., Biffi, A., Chan, L., et al. (2019). Pre-clinical Safety and Efficacy of Lentiviral Vector-Mediated Ex Vivo Stem Cell Gene Therapy for the Treatment of Mucopolysaccharidosis IIIA. Mol. Ther. Methods Clin. Dev. *13*, 399–413. https://doi.org/10.1016/j.omtm.2019.04.001.

39. Zonari, E., Desantis, G., Petrillo, C., Boccalatte, F.E., Lidonnici, M.R., Kajaste-Rudnitski, A., Aiuti, A., Ferrari, G., Naldini, L., and Gentner, B. (2017). Efficient Ex Vivo Engineering and Expansion of Highly Purified Human Hematopoietic Stem and Progenitor Cell Populations for Gene Therapy. Stem Cell Rep. *8*, 977–990. https://doi.org/10.1016/j.stemcr.2017.02.010.

40. Ferrari, G., Thrasher, A.J., and Aiuti, A. (2021). Gene therapy using haematopoietic stem and progenitor cells. Nat. Rev. Genet. *22*, 216–234. https://doi.org/10.1038/s41576-020-00298-5.

41. Poletti, V., Charrier, S., Corre, G., Gjata, B., Vignaud, A., Zhang, F., Rothe, M., Schambach, A., Gaspar, H.B., Thrasher, A.J., and Mavilio, F. (2018). Preclinical Development of a Lentiviral Vector for Gene Therapy of X-Linked Severe Combined Immunodeficiency. Mol. Ther. Methods Clin. Dev. *9*, 257–269. https://doi.org/10.1016/j.omtm.2018.03.002.

42. Cowan, M.J., Yu, J., Facchino, J., Fraser-Browne, C., Sanford, U., Kawahara, M., Dara, J., Long-Boyle, J., Oh, J., Chan, W., et al. (2022). Lentiviral Gene Therapy for Artemis-Deficient SCID. N. Engl. J. Med. *387*, 2344–2355. https://doi.org/10.1056/NEJMoa2206575.

43. Mesa-Nunez, C., Damian, C., Fernandez-Garcia, M., Diez, B., Rao, G., Schwartz, J.D., Law, K.M., Sevilla, J., Rio, P., Yanez, R., et al. (2022). Preclinical safety and efficacy of lentiviral-mediated gene therapy for leukocyte adhesion deficiency type I. Mol. Ther. Methods Clin. Dev. *26*, 459–470. https://doi.org/10.1016/j.omtm.2022.07.015.

44. Li, M., Morse, B., and Kassim, S. (2022). Development and Clinical Translation Considerations for the Next Wave of Gene Modified Hematopoietic Stem and Progenitor Cells Therapies. Expert Opin. Biol. Ther. *22*, 1177–1191. https://doi.org/10.1080/14712598.2022.2101361.

45. Carbonaro, D.A., Zhang, L., Jin, X., Montiel-Equihua, C., Geiger, S., Carmo, M., Cooper, A., Fairbanks, L., Kaufman, M.L., Sebire, N.J., et al. (2014). Preclinical demonstration of lentiviral vector-mediated correction of immunological and metabolic abnormalities in models of adenosine deaminase deficiency. Mol. Ther. *22*, 607–622. https://doi.org/10.1038/mt.2013.265.

46. Lu, J.Z., Hui, E.K.W., Boado, R.J., and Pardridge, W.M. (2010). Genetic engineering of a bifunctional IgG fusion protein with iduronate-2-sulfatase. Bioconjug. Chem. *21*, 151–156. https://doi.org/10.1021/bc900382q.

OMTM, Volume *32*

Supplemental information

Design and validation of a GMP stem cell manufacturing protocol for MPSII hematopoietic stem cell gene therapy

Stuart Ellison, Karen Buckland, Yuko Learmonth, Victoria Day, Spandan Kalra, Lauren Howe, Francisco José Roman-Rodriguez, Jose Bonafont, Laura Booth, Rebecca Holley, Jon Smythe, Simon Jones, Adrian Thrasher, Claire Booth, and Brian W. Bigger

Table S1. Current specification for batch release testing of a cryopreserved medicinal product for proposed clinical trial

Test	Method	Acceptance Criteria
Mycoplasma	PCR	Not Detected
Total Viable Cell Count/ml	Trypan Blue	0.5 to 17.5 x 10^6 TNC/ml
Total Viable Cell Count	Trypan Blue	Record
CD34+ Cell Purity and Identity	FACS	≥ 35% CD34+ cells
CD34+ Cell Dose	Calculated	Record
Cell Viability	Trypan Blue	≥ 70% viable cells
Sterility	BacTec	No Growth
Bacterial Endotoxins	Kinetic Turbidimetric	≤ 5 EU/kg/hr
Vector Copy Number	ddPCR	≥ 0.5 copy/cell

Table S2: Small scale hCD34+ stem cell transduction optimisation performed at GOSH. Low vector dose range.

**Methodology**	**Batch**	**MOI**	**Vector Conc. (x 10^8 IG/ml)**	**Healthy Donor Cells**	**Batch Testing**					
					**Test Attribute**	**Viability**	**CD34%**	**Clono-genicity**	**VCN (liquid culture)**	**Pass/ Fail**
					Method	Trypan Blue	Flow Cytometry	CFU assay	ddPCR	
					Acceptance Criteria	≥ 70%	For information only	≥ 4 CFUs/ 1000 cells plated	≥ 0.5	
GMP Experiment	21-SU-MPSII-01a	25	0.5	A		94.01	99.2	366	0.93	Pass
		12.5	0.25	A		92.87	99.0	346	0.85	Pass
		25	0.5	B		93.01	99.1	406	0.839	Pass
		12.5	0.25	B		92.94	99.2	374	0.827	Pass
		N/A	N/A	A		92.87	99.0	336	0.01	N/A
		N/A	N/A	B		99.6	99.6	398	0	N/A

Table S3: Small scale hCD34+ stem cell transduction optimisation performed at GOSH. High vector dose range.

*A minimum of 3.0 x 10^6 CD34+ cells/kg after transduction is required for infusion into the patient

Methodology	Batch	MOI**	Vector Conc. (x 10^8 IG/ml)*	Healthy Donor Cells	Batch Testing					
					**Test Attribute**	**Viability**	**CD34%**	**VCN (liquid culture)**	**VCN (pooled CFUs)**	**Pass/ Fail**
					Method	Trypan Blue	Flow Cytometry	ddPCR	ddPCR	
					Acceptance Criteria	≥ 70%	For information only	≥ 0.5	≥ 0.5	
GMP Experiment	21-SU-MPSII-01b	25	0.5	A		91.6	99.3	0.92	1.1	Pass
		50	1.0	A		89.13	99.5	1.19	1.08	Pass
		100	2.0	A		92.46	99.0	1.63	1.56	Pass
		25	0.5	C		91.07	99.2	0.99	1.5	Pass
		50	1.0	C		95.03	99.5	1.08	1.27	Pass
		100	2.0	C		92.53	99.5	1.92	2.09	Pass

*Cells from the same health donor A were used in both assays (table S2 and S3).**MOI = Multiplicity of infection

Table S4: UoM/Barnsley vs GOSH equivalent vector concentration comparison.

UoM/Barnsley MOI	GOSH MOI
25	18
50	35
100	71

Table S5. Individual VCN counts in single picked colonies from CFU assay, determined by QPCR. Left panel) Large scale transduction performed at Manchester research lab with LV IDS.ApoEII at MOI25 + TE with IL3 in the media, cultured in rectronection-coatedT175 flask. Right panel) 'At scale' transduction performed at NHSBT Barnsley research and development lab with LV IDS.ApoEII at MOI25 + TE with IL3 in the media, cultured in rectronection-coated T175 flask.

BFU-E	CFU-GM		BFU-E	CFU-GM
1.52	1.51		1.41	28.00**
5.96	6.42		1.89	1.10
1.90	1.88		1.34	6.28
4.92	4.49		2.26	10.24
3.86	1.35		1.25	8.43
3.56	5.73		2.69	3.51
1.51	4.55		4.27	2.41
6.42	2.76		10.20	8.07
1.88	1.31		0.15*	5.53
4.49	1.29		7.26	4.62
1.35	7.88		5.63	2.80
5.73	1.62		2.21	5.26
			3.58	4.07
			2.49	3.32
			4.06	0.82
			3.21	3.30
			1.28	-

* VCN below 0.3 classed as 0 (colony with no copies).
**VCN assay is accurate up to approximately 12 vector copies. VCNs above this cannot be accurately determined with this method.

Table S6. Overview of sampling timepoints and volumes taken for sterility testing performed for the NHSBT GMP validation runs.

Reagent / Sample	No. of Replicates/micro-organism	Inoculum volume per broth	Comments
Final product – CD11b-IDS.ApoEII LV Gene Modified Autologous CD34+ Cells in final container and condition as presented to patient	3	600µl	Cryopreserved in pilot vial 1% of final product volume
Fresh HPC Apheresis as collected from donor	1	2-4ml	1% of starting material volume depending on received volume
CD34- cell fraction post selection	1	5ml	1% of total selected cell product volumes (+ and – fractions)
Day 3 transduction media supernatent	1	5ml	>1% of total supernatant volume
Day 4 transduction media supernatent	1	5ml	>1% of total supernatant volume
Day 4 wash buffer supernatent	1	5ml	>1% of total supernatant volume
